# Supplementary material for: Reduced fish diversity despite increased fish biomass in a Gulf of California Marine Protected Area
Source: PeerJ. 2020 Apr 9;8:e8885. doi: 10.7717/peerj.8885 (PMC7151750; doi:10.7717/peerj.8885)
Supplement: Table S1 [file peerj-08-8885-s002.docx]

**Reduced fish diversity despite increased fish biomass in a Gulf of California marine protected area**

Georgina Ramírez-Ortiz, Héctor Reyes-Bonilla, Eduardo F. Balart, Damien Olivier, Leonardo Huato-Soberanis, Fiorenza Micheli, Graham J. Edgar.

**Electronic Supplementary Material: Tables and Figures**

**Table S1.** Number of censuses performed per year and per level of use in the different field periods, before the establishment of the MUMPA (2005-2007), after protection (2007-2014), and after the publication of the management plan (CONANP-SEMARNAT 2014) in PNZMAES.

|  |  |  | Level of use | | |
| --- | --- | --- | --- | --- | --- |
| Year | Time | Total census | No-take | Sustainable | Traditional |
| 2005 | Before | 11 | 4 | 4 | 3 |
| 2006 | Before | 22 | 8 | 8 | 6 |
| 2007 | After | 22 | 8 | 8 | 6 |
| 2008 | After | 22 | 8 | 8 | 6 |
| 2009 | After | 24 | 10 | 10 | 4 |
| 2010 | After | 30 | 12 | 12 | 6 |
| 2011 | After | 30 | 12 | 12 | 6 |
| 2012 | After | 30 | 12 | 12 | 6 |
| 2013 | After | 19 | 8 | 8 | 3 |
| 2014 | After | 30 | 12 | 12 | 6 |
| 2015 | Management plan | 29 | 11 | 12 | 6 |
| 2016 | Management plan | 30 | 12 | 12 | 6 |
| 2017 | Management plan | 21 | 9 | 8 | 4 |
