# Supplementary material for: Reduced fish diversity despite increased fish biomass in a Gulf of California Marine Protected Area
Source: PeerJ. 2020 Apr 9;8:e8885. doi: 10.7717/peerj.8885 (PMC7151750; doi:10.7717/peerj.8885)
Supplement: Table S2 — LMMs were performed and the results are presented in an ANOVA format. Variable names with a significant effect are bolded (based on the Satterthwaite df). Density and biomass variables were log-transformed (base 2). The effect of random variables, i.e. site, year, and season, are indicated by the Random Standard Deviance (RSD). [file peerj-08-8885-s003.docx]

**Table S2.** Comparison of reef fish diversity between the levels of use in PNZMAES. LMMs were performed and the results are presented in an ANOVA format. Variable names with a significant effect are bolded (based on the Satterthwaite df). Density and biomass variables were log-transformed (base 2). The effect of random variables, i.e. site, year, and season, are indicated by the Random Standard Deviance (RSD).

| Y ~ Use + (1\|Site) + (1\|Year) + (1\|Season) | | | | | |
| --- | --- | --- | --- | --- | --- |
|  | Variance | F | RSD Sites | RSD Years | RSD Seasons |
| Species richness | 4.03 | 0.19 | 2.4 | 2.05 | 1.44 |
| **Density** | 2.60 | 4.68 | 0.3 | 0.3 | 0 |
| Biomass | 2.06 | 1.62 | 0.3 | 0.3 | 0 |
| Functional richness | 0.001 | 0.19 | 0.03 | 0.03 | 0 |
| Funct. dispersion (D) | 0.005 | 1.17 | 0.05 | 0.02 | 0.1 |
| Funct. dispersion (B) | 0.018 | 2.48 | 0.019 | 0.019 | 0.01 |
| Funct. originality (D) | 0.003 | 1.48 | 0.04 | 0.01 | 0 |
| Funct. originality (B) | 0.001 | 0.24 | 0.05 | 0.05 | 0 |
