# Supplementary material for: Reduced fish diversity despite increased fish biomass in a Gulf of California Marine Protected Area
Source: PeerJ. 2020 Apr 9;8:e8885. doi: 10.7717/peerj.8885 (PMC7151750; doi:10.7717/peerj.8885)
Supplement: Table S3 — LMMs were performed and variable names with significant effects are bolded (based on the Satterthwaite df). Density and biomass were log-transformed (base 2). The effect of random variables, i.e. site and season, are indicated by the Random Standard Deviance (RSD). [file peerj-08-8885-s004.docx]

**Table S3.** Temporal analyses of reef fish diversity through a 13-year monitoring period in PNZMAES. LMMs were performed and variable names with significant effects are bolded (based on the Satterthwaite df). Density and biomass were log-transformed (base 2). The effect of random variables, i.e. site and season, are indicated by the Random Standard Deviance (RSD).

| Y ~ Year + (1\|Site) + (1\|Season) | | | | | | |
| --- | --- | --- | --- | --- | --- | --- |
|  | Intercept | Estimates | se | T | RSD Site | RSD Season |
| **Species richness** | 29.52 | -0.29 | 0.08 | -3.74 | 0.39 | 0.25 |
| Density | 8.24 | -0.03 | 0.01 | -1.93 | 0.42 | 0 |
| **Biomass** | 13.25 | 0.04 | 0.02 | 2.13 | 0.96 | 0 |
| **Functional richness** | 0.35 | -0.06*10^-1^ | 0.01*10^-1^ | -4.91 | 0.93 | 0 |
| **Funct. dispersion (D)** | 0.46 | 0.03*10^-1^ | 0.01*10^-1^ | 2.49 | 0.05 | 0.01 |
| **Funct. dispersion (B)** | 0.57 | -0.06*10^-1^ | 0.01*10^-1^ | -4.42 | 0.26 | 0.1 |
| Funct. originality (D) | 0.30 | 0.04*10^-3^ | 0.08*10^-2^ | 0.05 | 0.04 | 0 |
| Funct. originality (B) | 0.23 | 0.08*10^-2^ | 0.01*10^-1^ | 0.61 | 0.54 | 0 |
