# Supplementary material for: Reduced fish diversity despite increased fish biomass in a Gulf of California Marine Protected Area
Source: PeerJ. 2020 Apr 9;8:e8885. doi: 10.7717/peerj.8885 (PMC7151750; doi:10.7717/peerj.8885)
Supplement: Table S4 — Selection of the commercial species observed in our surveys was based on the list of commercial fish in PNZMAES (ESM1). The effect of random variables, i.e. site and season, are indicated by the Random Standard Deviance (RSD). [file peerj-08-8885-s005.docx]

**Table S4.** Temporal analyses of commercial fish biomass (log-transformed, base 2) through a 13-year monitoring period in PNZMAES. Selection of the commercial species observed in our surveys was based on the list of commercial fish in PNZMAES (ESM1). The effect of random variables, i.e. site and season, are indicated by the Random Standard Deviance (RSD).

| Y ~ Year + (1\|Site) + (1\|Season) | | | | | | |
| --- | --- | --- | --- | --- | --- | --- |
|  | Intercept | Estimates | se | T | RSD Site | RSD Season |
| Biomass | 12.04 | 0.026 | 0.025 | 1.04 | 0.36 | 0.29 |
