# Supplementary material for: Reduced fish diversity despite increased fish biomass in a Gulf of California Marine Protected Area
Source: PeerJ. 2020 Apr 9;8:e8885. doi: 10.7717/peerj.8885 (PMC7151750; doi:10.7717/peerj.8885)
Supplement: Table S5 — GLMMs with a binomial distribution were performed. Only species for which a significant effect was found, and the binned plot was respected, are shown. [file peerj-08-8885-s006.docx]

**Table S5.** Occurrence trend analyses of the common species through a 13-year monitoring period in PNZMAES. GLMMs with a binomial distribution were performed. Only species for which a significant effect was found, and the binned plot was respected, are shown.

| y~years+(1\|Sites) + (1\|Seasons) |  |  |  |
| --- | --- | --- | --- |
|  | Estimates | se | z |
| *Halichoeres chierchiae* | 0.106 | 0.034 | 3.09 |
| *Halichoeres dispilus* | 0.074 | 0.036 | 2.08 |
| *Scarus rubroviolaceus* | -0.072 | 0.035 | -2.06 |
| *Mycteroperca rosacea* | -0.078 | 0.038 | -2.05 |
| *Microspathodon dorsalis* | -0.086 | 0.040 | -2.14 |
| *Scarus ghobban* | -0.099 | 0.047 | -2.09 |
| *Sufflamen verres* | -0.132 | 0.041 | -3.21 |
| *Chromis limbaughi* | -0.216 | 0.046 | -4.74 |
| *Diodon holacanthus* | -0.278 | 0.046 | -6.06 |
