# Supplementary material for: Reduced fish diversity despite increased fish biomass in a Gulf of California Marine Protected Area
Source: PeerJ. 2020 Apr 9;8:e8885. doi: 10.7717/peerj.8885 (PMC7151750; doi:10.7717/peerj.8885)
Supplement: Table S6 — GLMMs with a negative binomial distribution were performed. Only species for which a significant effect was found are shown. Overdispersion values are also indicated (ratio of the residual deviance to the residual degrees of freedom). [file peerj-08-8885-s007.docx]

**Table S6**. Density trend analyses of the common species through a 13-year monitoring period in PNZMAES. GLMMs with a negative binomial distribution were performed. Only species for which a significant effect was found are shown. Overdispersion values are also indicated (ratio of the residual deviance to the residual degrees of freedom).

| Y ~ Year + (1\|Site) + (1\|Season) | | | | |
| --- | --- | --- | --- | --- |
|  | Estimates | se | z | Overdispersion |
| *Cephalopholis panamensis* | 0.130 | 0.022 | 6.002 | 0.859 |
| *Epinephelus labriformis* | 0.112 | 0.022 | 5.172 | 0.927 |
| *Balistes polylepis* | 0.084 | 0.038 | 2.194 | 1.017 |
| *Scarus compressus* | 0.065 | 0.030 | 2.204 | 1.479 |
| *Lutjanus argentiventris* | 0.055 | 0.025 | 2.253 | 0.914 |
| *Prionurus punctatus* | 0.047 | 0.024 | 2.002 | 1.350 |
| *Canthigaster punctatissima* | -0.024 | 0.010 | -2.370 | 0.987 |
| *Thalassoma lucasanum* | -0.027 | 0.013 | -2.070 | 0.859 |
| *Abudefduf troschelii* | -0.041 | 0.020 | -2.014 | 1.030 |
| *Johnrandallia nigrirostris* | -0.056 | 0.016 | -3.393 | 1.215 |
| *Microspathodon dorsalis* | -0.066 | 0.023 | -2.865 | 0.855 |
| *Stegastes rectifraenum* | -0.067 | 0.012 | -5.752 | 0.863 |
| *Scarus rubroviolaceus* | -0.080 | 0.031 | -2.579 | 0.922 |
| *Chromis limbaughi* | -0.107 | 0.033 | -3.215 | 1.307 |
| *Scarus ghobban* | -0.114 | 0.019 | -5.882 | 1.043 |
| *Chromis atrilobata* | -0.121 | 0.020 | -6.175 | 0.970 |
| *Diodon holocanthus* | -0.121 | 0.023 | -5.375 | 0.989 |
| *Cirrhitichthys oxycephalus* | -0.146 | 0.017 | -8.881 | 1.081 |
