# Supplementary material for: Reduced fish diversity despite increased fish biomass in a Gulf of California Marine Protected Area
Source: PeerJ. 2020 Apr 9;8:e8885. doi: 10.7717/peerj.8885 (PMC7151750; doi:10.7717/peerj.8885)
Supplement: Table S7 — GLMMs with a negative binomial distribution were performed. Only species for which a significant effect was found are shown. Overdispersion values are also indicated (ratio of the residual deviance to the residual degrees of freedom). [file peerj-08-8885-s008.docx]

**Table S7.** Biomass trend analyses of the common species through a 13-year monitoring period in PNZMAES. GLMMs with a negative binomial distribution were performed. Only species for which a significant effect was found are shown. Overdispersion values are also indicated (ratio of the residual deviance to the residual degrees of freedom).

| y~years+(1\|Sites) + (1\|Seasons) |  |  |  |  |
| --- | --- | --- | --- | --- |
|  | Estimates | se | z | Overdispersion |
|  |  |  |  |  |
| *Paranthias colonus* | 0.188 | 0.030 | 6.32 | 0.93 |
| *Epinephelus labriformis* | 0.179 | 0.052 | 3.42 | 0.28 |
| *Prionurus punctatus* | 0.124 | 0.025 | 4.94 | 1.22 |
| *Cephalopholis panamensis* | 0.106 | 0.048 | 2.20 | 0.33 |
| *Abudefduf troschelii* | 0.099 | 0.021 | 4.69 | 0.73 |
| *Bodianus diplotaenia* | 0.083 | 0.028 | 3.01 | 0.39 |
| *Scarus compressus* | 0.076 | 0.031 | 2.43 | 1.19 |
| *Holacanthus passer* | 0.071 | 0.021 | 3.34 | 1.07 |
| *Thalassoma lucasamum* | -0.092 | 0.044 | -2.10 | 0.10 |
| *Diodon holocanthus* | -0.110 | 0.044 | -2.51 | 0.24 |
| *Scarus rubroviolaceus* | -0.128 | 0.041 | -3.09 | 0.62 |
| *Chromis atrilobata* | -0.181 | 0.027 | -6.64 | 1.08 |
| *Mycteroperca rosacea* | -0.182 | 0.027 | -6.64 | 1.05 |
| *Cirritichthys oxycephalus* | -0.260 | 0.090 | -2.89 | 0.10 |
