# Supplementary figures and images for: Reduced fish diversity despite increased fish biomass in a Gulf of California Marine Protected Area
Source: PeerJ. 2020 Apr 9;8:e8885. doi: 10.7717/peerj.8885 (PMC7151750; doi:10.7717/peerj.8885)

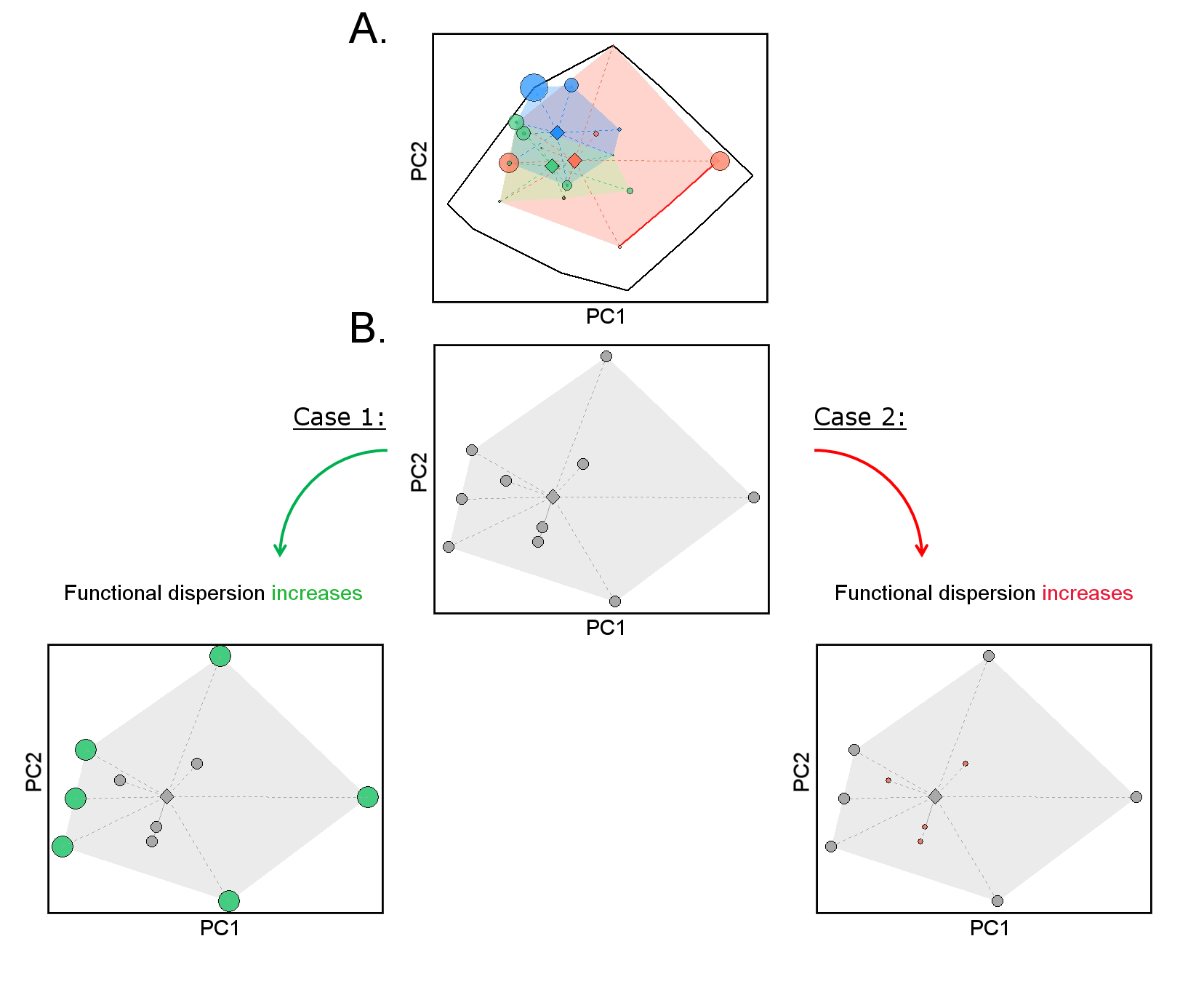

Supplement: Fifure S1 — (A) Illustration of the three functional indices used in this study. The colored-polygons represent the functional space that species present in three distinct transects encompass. The functional richness of a transect is the proportion of the functional space covered by the species present in the survey unit out of all the species pool (all transects). The dashed lines represent the distances between each species of a transect to the weighted centroid of the fish assemblage present in the transect. The weight can be the abundance or biomass of each species. The functional dispersion of a transect is the mean of these distances (weighted by abundance or biomass) divided by the half of the maximum distance among all the species pool. The thick red line represents the distance between a species and its nearest neighbor in one transect. The functional originality is the mean of these distances (weighted by abundance or biomass) divided by the maximum distance to the nearest neighbor found in all the species pool. (B) Two hypothetical cases where the functional dispersion increases. In the case 1, the increase in functional dispersion is due the increase in biomass or abundance of certain species (winners) located far from the centroid. In the case 2, the increase in functional dispersion is due to the decrease in biomass or abundance of certain species (losers) located close to the centroid. Functional dispersion can decrease for similar reasons. [file peerj-08-8885-s009.png]

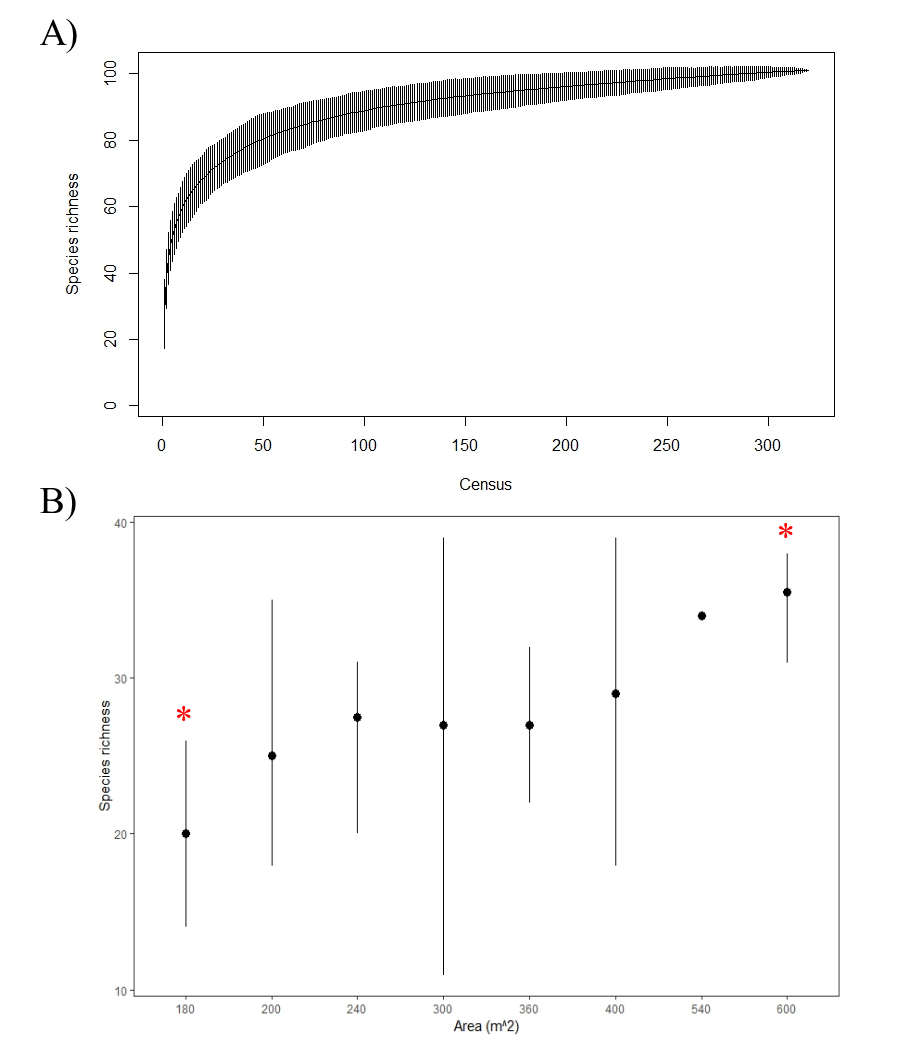

Supplement: Figure S2 — (A) Species accumulation curves (random method) for the study period (2005 to 2017) in PNZMAES. Independent variable is the number of census in the field period and dependent variable is the accumulated species as sampling effort increases. The figure shows that at around 50 censuses, the species pool was well represented. (B) Species richness per area sampled (average + standard deviation) at PNZMAES. Red asterisks exhibit significant differences between transect areas identified by a posteriori tests. [file peerj-08-8885-s010.png]

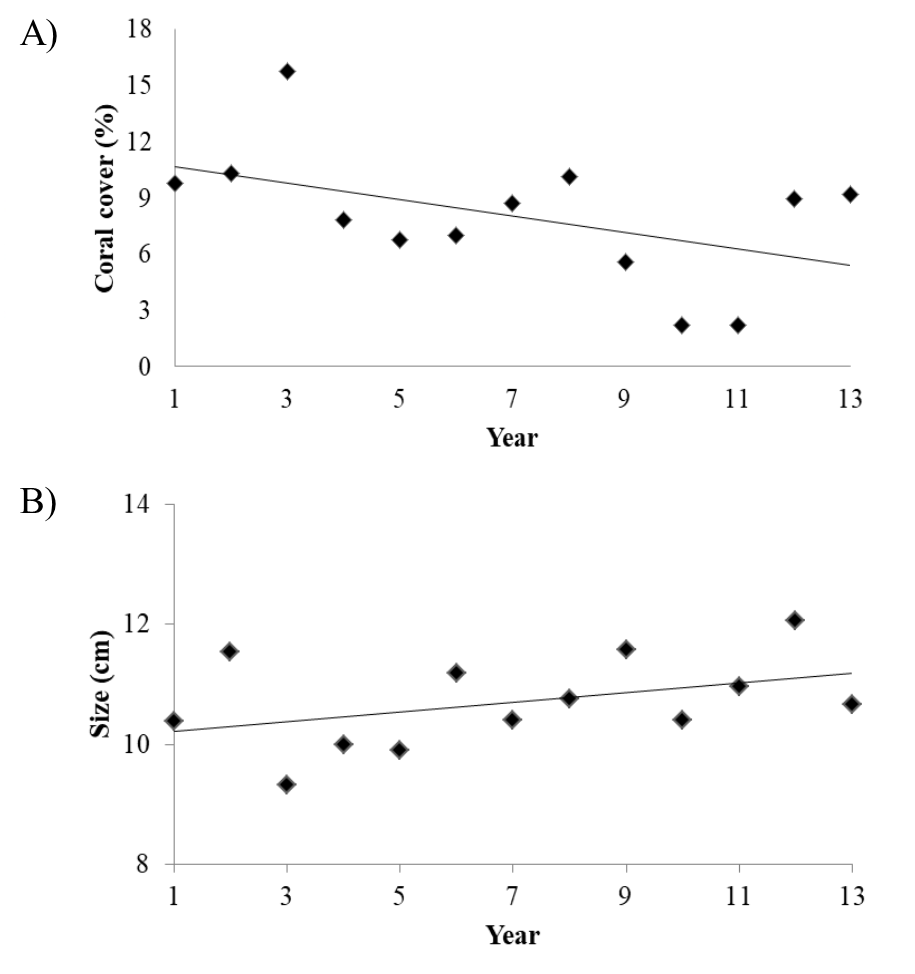

Supplement: Figure S3 — Each point represents the average value of each year, and the trend line for a linear regression analysis is displayed (Coral cover: y = -0.44x + 11.131, R2 = 0.23; Size: y = 0.081x + 10.13; R2 = 0.17). Non-published data provided by Niparajá. [file peerj-08-8885-s011.png]
